# Deficiency of GPI Glycan Modification by Ethanolamine Phosphate Results in Increased Adhesion and Immune Resistance of *Aspergillus fumigatus*

**DOI:** 10.3389/fcimb.2021.780959

**Published:** 2021-12-09

**Authors:** Haomiao Ouyang, Yi Zhang, Hui Zhou, Yubo Ma, Ruoyu Li, Jinghua Yang, Xiaowen Wang, Cheng Jin

**Affiliations:** ^1^ State Key Laboratory of Mycology, Institute of Microbiology, Chinese Academy of Sciences, Beijing, China; ^2^ Department of Dermatology and Venerology, Peking University First Hospital, Beijing, China; ^3^ Research Center for Medical Mycology, Peking University, Beijing, China; ^4^ Beijing Key Laboratory of Molecular Diagnosis on Dermatoses, Peking University First Hospital, Beijing, China; ^5^ National Clinical Research Center for Skin and Immune Diseases, Peking University First Hospital, Beijing, China

**Keywords:** *Aspergillus fumigatus*, GPI anchoring, adhesion, virulence, immune response, phosphoethanolamine, inflammation, macrophage killing

## Abstract

Glycosylphosphatidylinositol (GPI)-anchored proteins play important roles in maintaining the function of the cell wall and participating in pathogenic processes. The addition and removal of phosphoethanolamine (EtN-P) on the second mannose residue in the GPI anchor are vital for maturation and sorting of GPI-anchored proteins. Previously, we have shown that deletion of the *gpi7*, the gene that encodes an EtN-P transferase responsible for the addition of EtN-P to the second mannose residue of the GPI anchor, leads to the mislocalization of GPI-anchored proteins, abnormal polarity, reduced conidiation, and fast germination in *Aspergillus fumigatus.* In this report, the adherence and virulence of the *A. fumigatus gpi7* deletion mutant were further investigated. The germinating conidia of the mutant exhibited an increased adhesion and a higher exposure of cell wall polysaccharides. Although the virulence was not affected, an increased adherence and a stronger inflammation response of the mutant were documented in an immunocompromised mouse model. An *in vitro* assay confirmed that the Δ*gpi7* mutant induced a stronger immune response and was more resistant to killing. Our findings, for the first time, demonstrate that in *A. fumigatus*, GPI anchoring is required for proper organization of the conidial cell wall. The lack of Gpi7 leads to fast germination, stronger immune response, and resistance to macrophage killing.

## Introduction

*Aspergillus fumigatus* is a major human fungal pathogen causing invasive aspergillosis (IA). IA caused by *A. fumigatus* is initiated with inhalation of conidia. Upon inhalation, dormant conidia contact with airway epithelial cells, where the conidia adhere, swell, and germinate, which subsequently form hyphae and invade the lung and vessel of immunocompromised patients (Wasylnka and Moore, 2002; Wasylnka and Moore, 2003; Gomez et al., 2010; McCormick et al., 2010; Brown et al., 2012; Warris, 2014; Croft et al., 2016; Takahashi-Nakaguchi et al., 2018; Samalova et al., 2020). Despite the introduction of antifungal therapies, the mortality associated with this disease remains at least 50% (Herbrecht et al., 2002; Upton et al., 2007; Denning and Bromley, 2015). A better understanding of the pathogenesis of IA is required to develop novel therapeutic approaches.

Dormant conidia of *A. fumigatus* are covered with a hydrophobic rodlet layer consisting of the rodlet protein RodA and a pigment layer, which prevent the recognition of conidia by the host immune cells and confer resistance to killing by alveolar macrophages (Aimanianda et al., 2009; Fontaine et al., 2011; Bayry et al., 2014; Latgé and Beauvais, 2014; Latgé et al., 2017). During the swelling and germination process, the rodlet and melanin layers are gradually lost. As a result, the immunogenic cell wall components covered by rodlet and melanin layers, such as chitin, β-glucan, α-glucan, glycoproteins, and galactosaminogalactan (GAG), are exposed to immune recognition (Dague et al., 2008; Latgé and Beauvais, 2014; Valsecchi et al., 2019). Thus, the swelling and germination of conidia are not only required for the establishment of IA but also the activation of immune response.

Like other eukaryotes, *A. fumigatus* produces many glycosylphosphatidylinositol (GPI)-anchored proteins, which play important roles in maintaining the function of the cell wall, participating in polarized growth and thus the pathogenic process (Li et al., 2007; Yan et al., 2013; Ouyang et al., 2013; Ouyang et al., 2019; Samalova et al., 2020). Indeed, the GPI anchor is also thought as an ideal target for developing antifungal strategies and has drawn more attentions in recent years. The GPI anchor is synthesized in the endoplasmic reticulum (ER) with a series of multiple reactions and enzymes in most eukaryotes including fungi and mammals (Orlean and Menon, 2007; Kinoshita et al., 2008; Kinoshita, 2014). The maturation of the GPI anchor involves the addition of phosphoethanolamine (EtN-P) to mannose residues of the GPI glycan. In *Saccharomyces cerevisiae* and mammalian cells, the addition of EtN-Ps is catalyzed by EtN-P transferases MCD4/PIGN, GPI7, GPI13/PIGO, and GPI11/PIGF. Recent investigations reveal that Gpi7, Ted1, and p24 constitute a quality control system in the ER to prevent packaging of immature and misfolded GPI-anchored proteins into COPII vesicles (Reggiori et al., 1997; Fujita et al., 2009; Fujita and Kinoshita, 2012).

In *A. fumigatus*, we have previously shown that deletion of the *gpi7* gene blocks the addition of EtN-P to the second mannose residue and leads to a reduced amount of cell membrane GPI-anchored proteins, the mislocalization of the cell wall GPI-anchored protein Mp1, abnormal polarity, and autophagy. Also, the fast germination and reduced conidiation of the mutant are documented (Ouyang et al., 2019). These results suggest that Gpi7 is not only involved in the polarized growth of hyphae but also in the polarity establishment of conidia. However, little is known about the impact of GPI anchoring on the adherence and immune response of *A. fumigatus*. In this report, the adherence of germinating conidia of the Δ*gpi7* mutant was investigated. Also, the virulence and immune response of the mutant were tested.

## Results

### Adhesion of the Mutant Conidia on the Hydrophobic Surface

*Aspergillus fumigatus* conidial cell wall is covered by a rodlet layer and a melanin pigment layer (Aimanianda et al., 2009; Gastebois et al., 2009; Bayry et al., 2014). For swollen conidia and germlings, it has been known that exposure of polysaccharides in the conidial cell wall, including β-1,3-glucan, α-1,3-glucan, chitin, and galactomannan, is required for adherence to the host cell surface, extracellular matrix, and a variety of other substrates (Dague et al., 2008; Lamarre et al., 2009; Fontaine et al., 2010; Loussert et al., 2010; Sheppard, 2011; Gravelat et al., 2013; Valsecchi et al., 2019; Ball et al., 2020).

**Figure 1:**
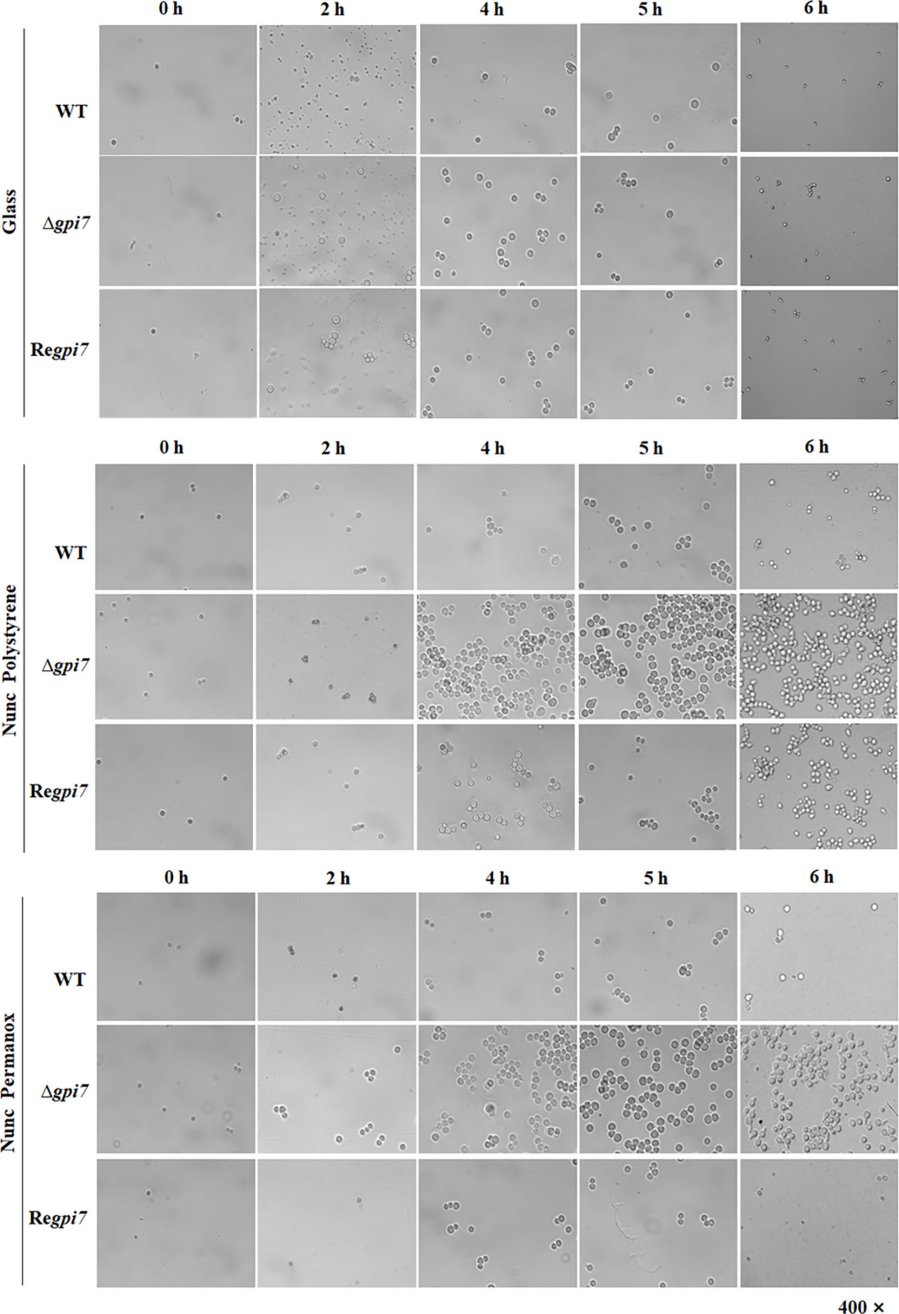
Adhesion of the Δ*gpi7* mutant to the hydrophobic surface. Conidia (1 × 10^9^) were added into 200 μl of CM medium sitting on a polystyrene cell culture slide or Permanox slide (Thermo Scientific Nunc). The slides were incubated at 37°C for 0–6 h. Non-adherent conidia were washed off with 0.1% Tween 20 in saline. The adherent conidia were washed with 1 ml 1% Tween 20 in saline, collected in an E-cup, and diluted with 0.1% Tween 20 in saline. One hundred microliters of each dilution was spread on CM plate and incubated at room temperature for 2 days and the number of colonies was counted. Glass slide was used as a non-hydrophobic surface. The experiment was repeated five times.

In order to assess the influence of GPI anchoring on the adhesive properties of *A. fumigatus*, the attachment of dormant, swollen, and germinating conidia to different surfaces was investigated. The conidia of the wild-type (WT), Δ*gpi7*, and Re*gpi7* strains were spotted onto microscopy slides made from polystyrene, Permanox, and glass. As can be seen in **Figure 1**, all dormant conidia were not able to adhere to glass, polystyrene, or Permanox. After 2–6 h of incubation, all adhered conidia on hydrophobic surfaces were swollen and germinating. As compared with the WT or Re*gpi7*, more mutant conidia adhered to the hydrophobic surface, indicating an increased adherence of the Δ*gpi7* mutant.

Usually conidial rodlet disappears after 5–6 h from the start of conidial swelling and germination (Ball et al., 2020). Under our experimental conditions, about 92% of the mutant conidia are germinated, while only 55% of the WT start germinating after incubation at 37°C for 6 h (Ouyang et al., 2019). As summarized in **Table 1**, after 6 h of incubation at 37°C, the adhered conidia of the mutant on Permanox and polystyrene were 29- and 15-fold than those of the WT, respectively. These results clearly demonstrate that the significant increase of adherence is due to the fast germination of the Δ*gpi7* mutant.

**Table 1:** Adhesion of the Δ*gpi7* mutant to the hydrophobic surface.

Strain	Glass (ns)	Polystyrene (***)	Permanox (***)
WT	(1.0 ± 0.4) × 10^2^	(1.8 ± 0.5) × 10^2^	(1.5 ± 0.4) × 10^3^
Δ*gpi7*	(1.2 ± 0.3) × 10^2^	(5.2 ± 0.7) × 10^3^	(2.3 ± 0.3) × 10^4^
Re*gpi7*	(1.1 ± 0.2) × 10^2^	(1.6 ± 0.5) × 10^3^	(1.7 ± 0.4) × 10^3^

Conidia (1 × 10^6^) were added into 200 μl of CM medium sitting on a polystyrene cell culture slide or Permanox slide (Thermo Scientific Nunc). The slides were incubated at 37℃ for 6 h. Non-adherent conidia were washed off with 0.1% Tween 20 in saline. The adherent conidia were washed with 1 ml 1% Tween 20 in saline, collected in an E-cup, and diluted with 0.1% Tween 20 in saline. One hundted microliters of each dilution was spread on the CM plate and incubated at room temperature for 2 days and the number of colonies was counted. The experiment was repeated five times. Mean and SD are presented. ns, not signifcant; ***, P < 0.001.

### Cell Wall of the Mutant Conidia

Gpi7 is involved in the transport and localization of GPI-anchored proteins required for cell wall organization, such as β-1,3-glucanosyltransferase Gel1, cell wall galactomannoprotein Mp1, and Ecm33 (Ouyang et al., 2019). To elucidate if Gpi7 affects the organization of conidial cell wall, we further determined the cell wall contents of the mutant conidia. As summarized in **Table 2**, the Δ*gpi7* mutant showed 57.6% increase of chitin and 20.5% increase of β-glucan as compared with the WT. Interestingly, the amount of glucosamine released from the cell wall proteins of the mutant increased by 85.4% though the content of the cell wall proteins extracted from the mutant conidia was similar with that from the WT. Also, galactose and mannose residues were detected in the mutant. These results indicate that glucosamine-containing polysaccharide, chitin, and β-glucan is increased in the mutant.

**Table 2:** Cell wall components of the mutant conidia.

Strain	Alkali soluble						Alkali insoluble	
	Glycoprotein (μg)					α-Glucan (μg)	β-Glucan (μg)	Chitin (μg)
	Protein (μg)	Glucosamine (μg)	Galactose (μg)	Mannose (μg)	Glucose (μg)			
WT	3,600.5 ± 7.5	8.0 ± 1.0	0	0	1.3 ± 0.2	19.8 ± 0.6	56.7 ± 1.4	8.4 ± 0.5
Δ*gpi7*	3,605 ± 12.5	14.9 ± 0.8	0.2 ± 0.1	0.7 ± 0.1	1.1 ± 0.1	21.4 ± 0.9	68.3 ± 1.1	13.3 ± 0.5
Re*gpi7*	3,710 ± 12	6.5 ± 0.8	0	0	0.7 ± 0.1	21.0 ± 0.6	57.0 ± 1.0	7.2 ± 0.4

Conidia (1 × 10^8^) were disrupted by glass beads using Disruptor Genie (Scientific Industries). The cell wall was collected by centrifugation and washed several times by distilled water. After washing, glycoprotein, α-glucan, β-glucan, and chitin were extracted from the cell walls. The contents of glycoprotein, α-glucan, β-glucan, and chitin were determined as described under Material and Methods. Three independent lyophilized conidia were used for cell wall analysis and the experiment was repeated three times.

**Figure 2:**
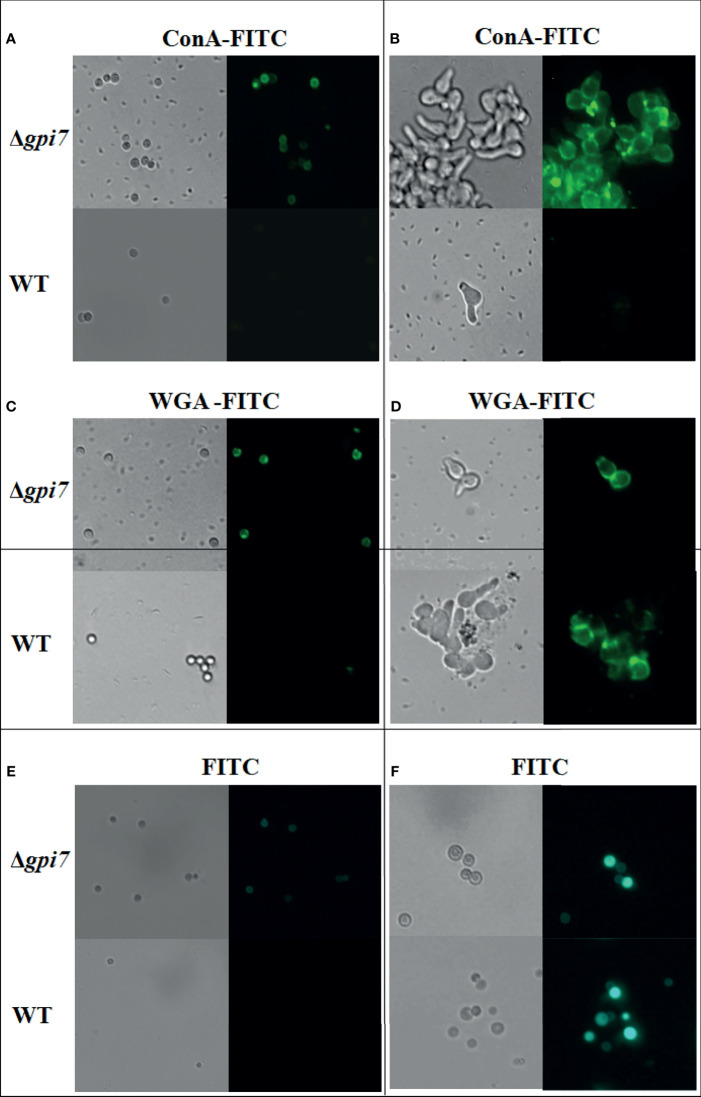
Detection of the dormant and germinating conidia with ConA and WGA. Dormant conidia **(A, C, E)** or 1 × 10^5^ conidia cultivated in 200 ml at 37°C for 5 h **(B, D, F)** were stained with ConA-FITC or WGA-FITC and then examined under a fluorescence microscope.

It has been shown that during germination of *A. fumigatus* cell wall, β-1,3-glucan and mannan are exposed to the conidial surface (Alsteens et al., 2013). When the dormant and germinating conidia were detected with ConA, a lectin specifically recognizes mannose and glucose, both dormant and germinating conidia of the mutant were positively stained by FITC-labeled ConA (**Figures 2A, B** **)**, indicating an increased exposure of cell wall polysaccharides on the surface of dormant and germinating conidia of the mutant. When conidia were detected with FITC-labeled WGA, a lectin binds N-acetylglucosamine (GlcNAc), the dormant conidia of the mutant exhibited an increased positive staining as compared with the WT (**Figure 2C**), while the germinating conidia of the mutant were similar with the WT (**Figure 2D**). When both dormant and germinating conidia were detected with FITC, no difference was observed between the WT and mutant (**Figures 2E, F**). These results suggest that the lack of Gpi7 affects the organization of the conidial cell wall and results in an increased exposure of cell wall polysaccharides in the dormant mutant conidia and fast exposure of mannose- and glucose-containing polysaccharides during germination of *A. fumigatus*.

**Figure 3:**
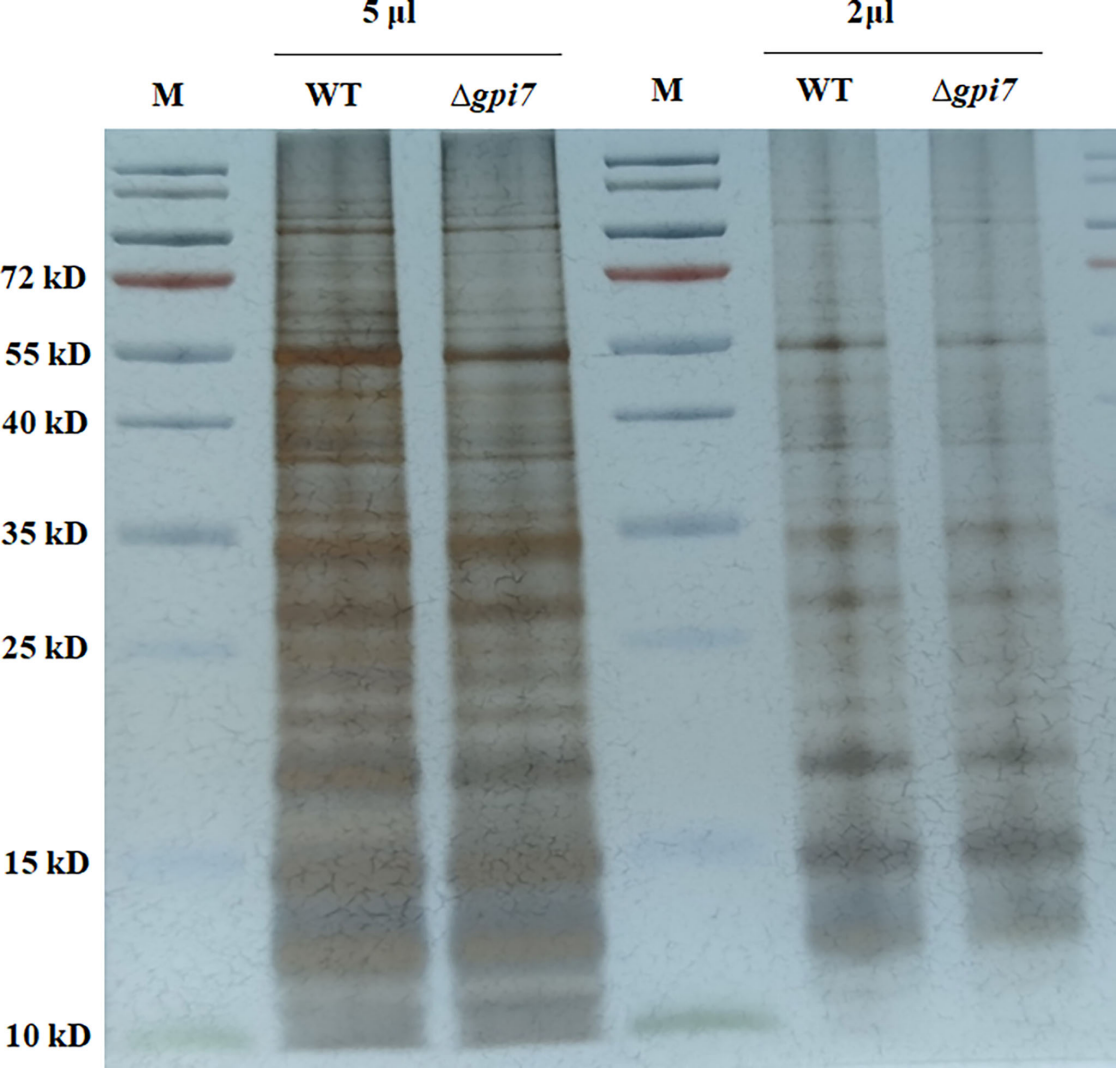
Detection of the hydrophobin rodlet in the mutant conidia. Conidia were subjected to sonication and low-speed centrifugation. The supernatant was ultracentrifuged for 1 h at 50,000×*g*. The pellet was boiled in SDS-PAGE loading buffer and washed twice with loading buffer and three times with distilled water. The resulting pellet was lyophilized and subsequently treated with 100% trifluoroacetic acid (TFA) for 10 min at room temperature. After removal of the acid under a stream of nitrogen at room temperature, the pellet was dissolved in SDS-PAGE loading buffer, boiled for 15 min, and subjected to SDS-PAGE (15% polyacrylamide). The gel was visualized by silver staining.

*Aspergillus fumigatus* dormant conidia are covered by a non-covalently attached hydrophobin rodlets (Templeton et al., 1995; Valsecchi et al., 2019). To define the mechanism of the increased exposure of the cell wall polysaccharides in the mutant, we further determined the rodlet of the mutant by using the protocol described by Paris et al. (2003). As shown in **Figure 3**, the surface proteins extracted from the mutant were similar with those from the WT, indicating that the content of RodA was not affected in the mutant.

Taken together, it is likely that the increased adherence of the mutant is contributed by fast germination, which led to the fast exposure of increased cell wall mannose- and glucose-containing polysaccharides of the mutant.

As we observed changes in the conidial cell wall components of the mutant, we further checked its cell wall integrity by determining the survival rate of the dormant mutant conidia in water. As a result, 40% of the mutant conidia lost their viability after 8 weeks of incubation in water at room temperature in comparison with 90% viability of the WT ones (**Figure S1A**). Interestingly, the viability of the mutant conidia remained 60% after 4 weeks of incubation in water at 42°C, whereas the viability of the WT conidia was only 40% (**Figure S1B**), which suggest a better temperature tolerance of the mutant conidia.

### Virulence of the Mutant in an Immunocompromised Mouse Model

**Figure 4:**
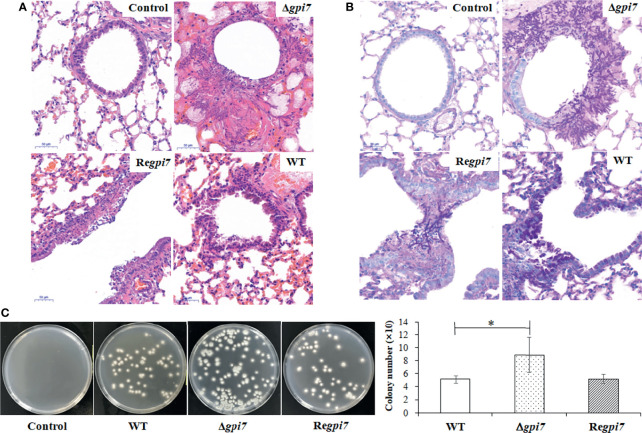
Lung tissue of immunosuppressed mice infected by the mutant. Virulence of the WT, Δ*gpi7*, and Re*gpi7* strains was detected with immunosuppressed mice. In **(A)**, the right lung from each mouse was dissected at day 3 post-infection and fixed in 4% (v/v) paraformaldehyde in physiological saline. In **(B)**, lung sections were stained with hematoxylin–eosin (HE) and periodic acid-Schiff (PAS). In **(C)**, the lung from each mouse was homogenized by OSE-Y30 (TIANGEN) at day 1 post-infection, and the number of conidia was counted by flat dilution counting. The experiment was repeated five times for each strain. Results are presented as mean ± SD. *P < 0.05.

To evaluate the contribution of the increased adherence on the virulence of the Δ*gpi7* mutant, freshly harvested conidia from the WT, Δ*gpi7*, and Re*gpi7* strains were inoculated into immunocompromised mice. The mice were monitored for 30 days after inoculation. Although no significant difference in mortality was documented between the WT and the Δ*gpi7* mutant, IA was observed in the lung tissues of mice inoculated with the mutant conidia at day 3 post-inoculation (**Figure 4A**). The histological feature of mice infected with the Δ*gpi7* mutant was observed with necrosis encompassed with numerous neutrophils and macrophages, while neutrophilic infiltration and necrosis were much gentler in the WT, indicating that the Δ*gpi7* mutant can stimulate stronger inflammatory response than the WT. The lung sections were also stained with periodic acid-Schiff stain, and the invasive hyphae of the mutant could be easily observed in the bronchial tubes and alveoli (**Figure 4B**). It was also noted that the number of conidia of the Δ*gpi7* mutant was much larger than that of the WT (**Figure 4B**).

After 24 h post-inoculation, the lung tissue was separated from immunosuppressed mice and then ground. The *A. fumigatus* conidia in ground lung tissue were washed out and counted. As shown in **Figure 4C**, the conidia in the lung infected by the Δ*gpi7* mutant were 2-fold of those from the WT or Re*gpi7*. These results indicate an increase of adhesion to the lung cells and a resistance to killing of the Δ*gpi7* mutant.

### Immune Response of the Δgpi7 Mutant

**Figure 5:**
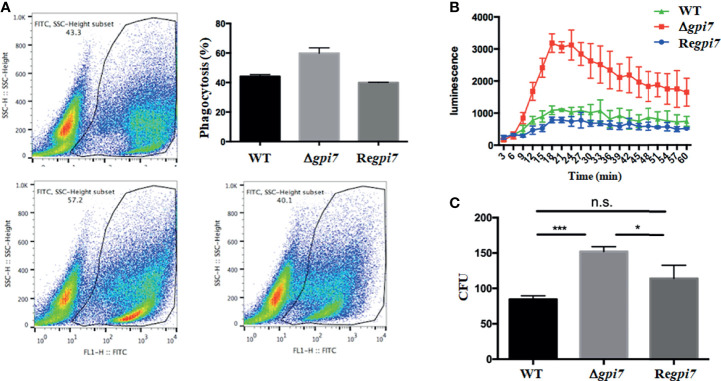
*In vitro* assay of immune response of the mutant. In **(A)**, phagocytosis of the FITC-labeled WT, Δ*gpi7*, and Re*gpi7* conidia by THP-1−derived macrophages was determined using flow cytometry; in **(B)**, human PMNs were stimulated with alive WT, Δ*gpi7*, or Re*gpi7* conidia and intracellular ROS produced by PMNs was measured in the presence of luminol and chemiluminescence; and in **(C)**, survival of *A. fumigatus* conidia in immune cells was determined by counting the CFUs after 2 h of incubation of THP−1−derived macrophages with conidia. The bars represent mean ± SD from at least three independent experiments. Data were analyzed by unpaired *t*-test. *P < 0.05, ***P < 0.001; n.s., not significant.

To assess the immune response of the dormant conidia of the Δ*gpi7*, phagocytic ratio and ROS production were measured. As shown in **Figure 5A**, at 2 h post-incubation with THP−1−derived macrophages, the phagocytic ratio of the Δ*gpi7* strain was significantly higher than that of the WT or Re*gpi7* strain. Although a previous study has shown that the mutant conidia germinate 2 h earlier than the WT, it should be pointed out that none of the WT or mutant conidia germinated after incubation at 37°C within 2 h (Ouyang et al., 2019). As shown in **Figure 1**, only a few conidia of the WT or mutant were swollen after incubation at 37°C for 2 h, and no significant difference was observed between the WT and mutant. Therefore, we postulate that the increased phagocytosis is contributed by the exposure of polysaccharides on the surface of the dormant mutant conidia. In addition, an intracellular ROS production assay was carried out by co-culture of the mutant conidia with human polymorphonuclear neutrophils (PMNs) at 37°C for 1 h. As shown in **Figures 5B, C**, the neutrophils exposed to the Δ*gpi7* strain produced higher intracellular ROS than those exposed to the WT or Re*gpi7* strain. Collectively, these results demonstrate that the Δ*gpi7* dormant conidia can induce stronger immune response *in vitro*.

To detect whether Gpi7 influences the survival capacity of *A. fumigatus* in immune cells *in vitro*, colony-forming units (CFUs) were determined after 2 h of incubation of *A. fumigatus* conidia with THP−1−derived macrophages. As was noticed, CFUs recovered from the THP−1−derived macrophages infected by the Δ*gpi7* strain were significantly higher than other strains, which indicates a resistance to killing of the Δ*gpi7* strain and is consistent with the higher fungal loads of the Δ*gpi7* mutant in the lung of infected mice.

## Discussion

Adherence of *A. fumigatus* to host constituents is thought to be an early and critical step in the initiation of colonization and infection (Sheppard, 2011). Inhibition of these adherence events may provide a useful therapeutic strategy to reduce morbidity and mortality from *A. fumigatus*-mediated disease (Gravelat et al., 2013).

In immunocompromised hosts, IA is initiated with the inhalation of airborne conidia. Upon inhalation, *A. fumigatus* conidia contact with airway epithelial cells or pulmonary macrophages where they adhere before initiating germination and hyphal growth (Wasylnka and Moore, 2002; Wasylnka and Moore, 2003; Wasylnka et al., 2005; Gomez et al., 2010). Although the dormant conidia first come in contact with host airway epithelial cells after inhalation, only two proteins, AFUA_4G01030 (hypothetical protein) and AFUA_4G08805 (hemolysin‐like protein), have been identified as adhesins of the dormant conidia (Takahashi-Nakaguchi et al., 2018). Little is known of the molecular mechanisms underlying the adherence of *A. fumigatus* dormant conidia to host pulmonary epithelial cells.

In contrast to the dormant conidia, the adhesion of swollen and germinating conidia of *A. fumigatus* has been extensively studied. Accumulated lines of evidence have shown that polysaccharides in the conidial cell walls are required for adherence to the host cell surface, extracellular matrix, and a variety of other substrates. During germination, conidia lose their rodlet layers and α-1,3-glucan moves from the inner layer to the conidial surface, which causes an increase in adhesive properties and interacts with phagosome biogenesis (Fontaine et al., 2010; Beauvais et al., 2013). Later, the appearance of GAG on the cell wall surface functions as the dominant adhesin of *A. fumigatus* and mediates adherence to plastic, fibronectin, and epithelial cells (Fontaine et al., 2011; Gravelat et al., 2013; Robinet et al., 2014; Lee et al., 2015).

In this study, we found that adherence of the swollen and germinating conidia of the Δ*gpi7* mutant was increased on hydrophobic surface. Further analysis revealed an increase of the cell wall polysaccharides and an exposure of the polysaccharides on the surface of the dormant mutant conidia. However, the content of rodlet proteins of the dormant mutant conidia was similar with the WT. These results suggest that the increased adhesion of the mutant conidia is contributed by the exposure of the increased cell wall polysaccharides, such as β-glucan- and glucosamine-containing polysaccharides. On the other hand, we have shown that about 55% of the mutant conidia germinate while only 5% of the WT start germinating after 4 h of incubation at 37°C (Ouyang et al., 2019). Therefore, under our experimental conditions, the fast germination should be also another important factor that contributes to the increased adhesion of the mutant. Taken together, it is reasonable to conclude that the increased adhesion associated with the mutant is attributed to either an increase of cell wall polysaccharides or fast germination associated with the mutant.

Rodlet and melanin layers are known to prevent the recognition of conidia by the host immune cells and confer resistance to killing (Aimanianda et al., 2009; Fontaine et al., 2011; Bayry et al., 2014). During germination, cell wall β-1,3-glucan, α-1,3-glucan, chitin, and galactomannan are exposed to immune cells to induce or suppress immune response (Aimanianda et al., 2009; Gastebois et al., 2009; Bayry et al., 2014; Wong et al., 2020). In our study, the mutant conidia did not result in an increased virulence in an immunocompromised mouse model; however, a stronger inflammation response was observed in mice infected with the mutant. As β-1,3-glucan is recognized by Dectin-1 and induces inflammation (Brown and Gordon, 2001; Drummond and Brown, 2011; Keizer et al., 2020), the stronger inflammation response associated with the mutant can be ascribed to the increase of β-1,3-glucan in conidial cell wall.

It is believed that fast swelling and germinating conidia can be more efficiently cleared by the immune system, while slow swelling and germinating conidia seem to be taken up more efficiently by epithelial cells and thereby hide from the immune system (Rosowski et al., 2018; Keizer et al., 2020). In contrast to these previous reports, we found more conidia in the mouse lung infected by the Δ*gpi7* mutant, suggesting that the fast swelling and germinating conidia of the mutant are resistant to killing by innate immune cells.

In the immunocompetent host, alveolar macrophages and neutrophils are two major innate immune cells involved in *A. fumigatus* clearance. Upon inhalation of *A. fumigatus* conidia, alveolar macrophages rapidly internalize but slowly kill *A. fumigatus* conidia inside acidified phagolysosomes (Latgé et al., 2017). As essential immune effector cells against *A. fumigatus*, neutrophils utilize an array of oxidative and non-oxidative mechanisms to combat the different infectious stages of the fungus (Mircescu et al., 2009). Using a cellular model, we found that the Δ*gpi7* conidia exhibited an increased phagocytic ratio in THP-1−derived macrophages and an increase of ROS induction in neutrophils, which is consistent with the stronger inflammatory infiltrations in the lung of the mice infected with the Δ*gpi7* mutant. This enhanced immune response is probably led by the exposure of GlcNAc-, glucosamine-, glucose-, and mannose-containing polysaccharides on the surface of the Δ*gpi7* conidia, which are known as typical fungal pathogen-associated molecular patterns (PAMPs). Therefore, host immune cells can recognize the mutant conidia more efficiently with their pattern recognition receptors (PRRs) and initiate the downstream immune responses including phagocytosis and ROS production. Moreover, we found that the survival rate of the Δ*gpi7* mutant in THP-1−derived macrophages was higher than that of the WT, which is in accordance with the higher fungal loads of the Δ*gpi7* mutant in the lung from infected mice. As GAG can suppress host inflammatory responses by mediating resistance to NADPH oxidase-dependent neutrophil killing and increased resistance to neutrophil extracellular traps (Gravelat et al., 2013; Lee et al., 2015), it is likely that exposure of glucosamine-containing polysaccharides on the surface of the Δ*gpi7* conidia is responsible for stronger resistance against cytotoxic damage from the phagocytic cells.

In conclusion, Gpi7 is required for the organization of the inner cell wall of conidia and affects conidial germination of *A. fumigatus*. Deletion of the *gpi7* results in an increase in cell wall polysaccharides, an exposure of polysaccharides on the conidial surface, and a faster germination. Faster and more exposure of the cell wall components enhances the adherence of the Δ*gpi7* mutant to the hydrophobic surface. Using an immunocompromised mouse model, for the first time, we show that fast germination and exposure of the increased cell wall polysaccharides of the Δ*gpi7* mutant lead to an increased adhesion to the lung cells, stronger inflammation response, and resistance to macrophage killing. Additionally, we show that exposure of polysaccharides on the conidial surface of the Δ*gpi7* mutant can induce stronger immune response *in vitro* by stimulating more efficient internalization by macrophages and resistance to killing by neutrophils.

## Material and Methods

### Strains and Growth Conditions

*Aspergillus fumigatus* WT, Δ*gpi7*, and Re*gpi7* strains used in this study were described previously (Ouyang et al., 2019)*. Aspergillus fumigatus* strain was grown for 2 days at 37°C on complete medium (CM) (Cove, 1966). Conidia were harvested from solid CM medium with 0.1% Tween 20. The concentration of conidia was measured by hemocytometer counting.

### Cell Adhesion Analysis

Spore solutions of the WT, Δ*gpi7*, and Re*gpi7* strains were prepared in 0.1% Tween 20 in saline at a concentration of 1 × 10^8^ conidia/ml. Ten microliters of each spore solution was added into 200 μl of CM medium sitting on a microscopy slide and mixed well by pipetting and stirring. Two different hydrophobic surfaces were used: polystyrene cell culture slides and Permanox slides (Thermo Scientific Nunc). Ordinary glass microscopy slides were used as a non-hydrophobic surface. The slides were incubated at 37°C for 0–6 h, the culture medium was removed, the slides were washed shortly in 0.1% Tween 20 in saline, and the adherent spores were removed by running 1 ml 1% Tween 20 in saline over the slide. For each slide, this solution was collected in an E-cup, appropriate dilutions in 0.1% Tween 20 in saline were prepared, and 100 μl of each dilution was spread on the CM plate and incubated at room temperature for 2 days, after which the number of colony-forming units was determined. For statistical significance, each experiment was performed five times for each strain and condition.

### Chemical Analysis of the Cell Wall

Conidia (1 × 10^8^) were washed with deionized water and disrupted by 0.2 g of glass beads (0.5 mm diameter) containing 50 mM NH_4_HCO_3_ at pH 8.0. The conidia was broken by Disruptor Genie (Scientific Industries) 15 times for 5 min each time. Then, the cell homogenates were centrifuged and washed several times by distilled water. Three independent samples of lyophilized conidia were used for cell wall analysis, and the experiment was repeated three times. After washing, cell walls were treated with 1 M KOH and incubated at 70°C for 30 min to release glycoprotein and α-glucan. The alkali-soluble materials were acidified with acetic acid to pH 5.0, and the precipitated α-glucans were collected by centrifugation and washed with water. The glycoprotein in the supernatant was precipitated with 2 volumes of ethanol, washed twice with 64% ethanol, and dissolved in distilled water. The glycoprotein concentration was determined using the Lowry protein assay (Takaku et al., 2010). Monosaccharides were liberated from glycoproteins by acid hydrolysis (6 M HCl at 100°C for 2 h) and separated on a CarboPac PA1 anion-exchange column, equipped with an Amino Trap guard column. Elution was performed at room temperature at a flow rate of 1 ml/min with 18 mM NaOH. The alkali-insoluble materials were washed with water several times and digested in 6 M HCl at 100°C for 2 h to release monosaccharides from β-glucan and chitin. After digestion, HCl was evaporated and the residues were dissolved in 0.2 ml distilled water (Yamashita et al., 1999; Lamson et al., 2002; Guo et al., 2015). The amounts of α-glucan and β-glucan present were estimated by measuring released glucose using the phenol/sulfuric acid method (Zhang et al., 2016). Chitin content was determined by measuring the N-acetylglucosamine released after digestion (McMillan et al., 1999).

### Conidia Staining

Spores (1 × 10^5^) were inoculated in 200 ml of CM and incubated at 37°C. The resting spores and swollen conidia were separately stained with ConA-FITC or WGA-FITC and then examined under a fluorescence microscope.

### Extraction and Electrophoresis of the Rodlet

The rodlet layer was extracted as described by Paris et al. (2003). Briefly, the conidia were subjected to sonication. After removal of the remaining conidia by low-speed centrifugation, the supernatant was ultracentrifuged for 1 h at 50,000×*g*. The pellet was boiled in SDS-PAGE loading buffer and then washed twice with SDS-PAGE loading buffer and three times with distilled water. The resulting pellet was lyophilized. The lyophilized material was subsequently treated with 100% trifluoroacetic acid (TFA) for 10 min at room temperature. Then, the acid was removed under a stream of nitrogen. Dried extracts were dissolved in SDS-PAGE loading buffer and incubated in boiling water for 15 min. Proteins were subjected to SDS-PAGE (15% polyacrylamide) and visualized by silver staining.

### Conidia Survival

The conidia (1 × 10^5^) were kept on distilled water for different storage times (1, 2, 4, 8 weeks) at different temperatures (room temperature and 42°C) separately. To calculate the spore survival, the conidia were inoculated on CM and cultured at 37°C. The germination of conidia was counted. For statistical significance, each experiment was performed five times for each strain.

### THP-1 Cells

The human monocyte cell line—THP−1 cells—was cultured in RPMI 1640 (Gibco, Thermo Fisher Scientific, Inc., Waltham, MA, USA) containing 10% fetal bovine serum (FBS, Gibco, Thermo Fisher Scientific) and 1% penicillin–streptomycin antibiotic mixture at 37°C with 5% CO_2_. To prepare THP−1−derived macrophages, THP−1 cells (1 × 10^6^/ml) were plated onto six-well plates and incubated with phorbol 12-myristate 13-acetate (PMA) at a concentration of 50 ng/ml for 48 h. After incubation, adherent macrophages were maintained in complete medium at 37°C with 5% CO_2_ and utilized in phagocytosis and killing experiments (Shabani et al., 2020; Qin et al., 2020).

### Assay for Conidial Killing

THP−1−derived macrophages (1 × 10^6^/ml) were exposed to resting conidia of the WT, Δ*gpi7*, and Re*gpi7* strains (MOI = 10) in six−well plates at 37°C and 5% CO_2_ for 2 h, respectively. At 2 h time point, non−adherent cells and non−phagocytosed conidia were removed by washing the cells three times with PBS. To lyse the cells and harvest the conidia, sterile water was added and mixed vigorously with standing for 5 min. Cellular lysis was confirmed by microscopy. The serial dilutions were performed in sterile water and immediately plated on potato dextrose agar (PDA; BD Biosciences, San Jose, CA, USA). Colonies were counted following incubation for 24 h at 37°C (Liu et al., 2017).

### Measurement of Conidia Phagocytosis

To prepare fluorescein isothiocyanate (FITC)−labeled conidia, a total of 1 × 10^7^/ml conidia were suspended in 100 ml 0.05 M sterile carbonate–bicarbonate buffer (cat. no. C3041; Sigma−Aldrich; Merck KGaA) (pH 9.6). After 10 mg FITC powder was dissolved in 1 ml DMSO, the FITC solution was quickly added to the above carbonate–bicarbonate solution containing 1 × 10^7^/ml conidia, and then the solution was stirred with a magnetic stirrer about 1 h at 4°C in the dark. The suspension was then washed three times with PBS. The FITC−labeled conidia were resuspended in PBS and adjusted to the desired concentration (1 × 10^9^/ml). The FITC-labeled conidia were confirmed by a fluorescence microscope.

THP−1−derived macrophages (1 × 10^6^/ml) were co−cultured with FITC−labeled resting conidia of WT, Δ*gpi7*, and Re*gpi7* strains (MOI = 10) in 2 ml complete RPMI 1640 medium at 37°C and 5% CO_2_ for 2 h, respectively. The supernatants were discarded and the wells were washed gently three times with ice-cold PBS. THP−1−derived macrophages were lifted from the wells with gentle pipetting with wash buffer. To define the ingested conidia, THP−1−derived macrophages were further stained with FITC-conjugated anti-human CD11b antibody. Data were acquired on a BD FACSCalibur system and analyzed with the FlowJo 7.6 software (Marr et al., 2001).

### Neutrophil and ROS Assays

Human PMNs were isolated from whole blood specimens (three healthy donors) by density-gradient centrifugation using the Ficoll-Paque Plus (GE Healthcare, USA) as described previously (Voyich et al., 2005). PMN was resuspended in 1 ml HBSS and adjusted to the concentration of 2 × 10^6^/ml. Using a 96-well flatbottom plate, 50 μl PMN (2 × 10^6^/ml), 50 μl 40% FBS, and 50 μl conidia (4 × 10^7^/ml) were put together, with 50 μl luminol solution added finally. Chemiluminescence was measured at 37°C for 60 min in 3 min intervals in an automated LB96V MicroLumat Plus luminometer (EG&G Berthold, Germany) (Guerra et al., 2016).

### Mice Virulence

Virulence of the WT, Δ*gpi7*, and Re*gpi7* strains was detected with immunosuppressed mice (Latgé, 1999; Li et al., 2007; Sugui et al., 2007; Li et al., 2012). Briefly, four groups (control, WT, Δ*gpi7*, and Re*gpi7*) of strains each containing 20 male BALB/c mice (18–20 g) were used for the virulence experiments. Fresh conidia were washed from CM plates and suspended in 0.01% Tween 20 in saline with inoculum of 3 × 10^5^ CFU/g mouse weight in 30 μl volume. Mice were immunosuppressed by injection with 150 mg/kg mouse weight cyclophosphamide on days −3, −1, +3, +6, and +9 and 200 mg/kg mouse weight hydrocortisone on day −1. Mice were inoculated with conidia by nasal feeding on day 0 and monitored twice each day for 30 days after inoculation and mortality was recorded. Mice surviving in the experiment were humanely terminated on day 30. The lung from each mouse was homogenized by OSE-Y30 (TIANGEN) at day 1 post-infection. The conidia were washed out from the same amount of homogenate, diluted, and counted by flat dilution counting. The right lung from each mouse was dissected at day 3 post-infection and fixed in 4% (v/v) paraformaldehyde in physiological saline. Sections were stained with hematoxylin–eosin (HE), and periodic acid-Schiff (PAS) by standard techniques. For statistical significance, each experiment was performed five times for each strain.

## Data Availability Statement

The original contributions presented in the study are included in the article/**Supplementary Material**. Further inquiries can be directed to the corresponding authors.

## Ethics Statement

The animal study was reviewed and approved by Peking University First Hospital, Peking University.

## Author Contributions

CJ, HO, and XW conceived the study. HO, YZ, and YM performed the biochemical experiments and virulence analysis. HO, YZ, HZ, RL, JY, XW, and CJ analyzed and interpreted the data. CJ, HO, and XW wrote the manuscript with input from all authors. All authors contributed to the article and approved the submitted version.

## Funding

This work was supported by the National Natural Science Foundation of China (31630016) to CJ.

## Conflict of Interest

The authors declare that the research was conducted in the absence of any commercial or financial relationships that could be construed as a potential conflict of interest.

## Publisher’s Note

All claims expressed in this article are solely those of the authors and do not necessarily represent those of their affiliated organizations, or those of the publisher, the editors and the reviewers. Any product that may be evaluated in this article, or claim that may be made by its manufacturer, is not guaranteed or endorsed by the publisher.
