# Supplementary material for: Deficiency of GPI Glycan Modification by Ethanolamine Phosphate Results in Increased Adhesion and Immune Resistance of Aspergillus fumigatus
Source: Front Cell Infect Microbiol. 2021 Dec 9;11:780959. doi: 10.3389/fcimb.2021.780959 (PMC8695850; doi:10.3389/fcimb.2021.780959)
Supplement: Supplementary file 1 [file DataSheet_1.pdf]

Supplemental data

Deficiency of GPI glycan modification by ethanolamine phosphate results in increased adhesion and immune resistance of *Aspergillus fumigatus*

Haomiao Ouyang[1#]*, Yi Zhang[2#], Hui Zhou[1], Yubo Ma[2], Ruoyu Li[2], Jinghua Yang[1], Xiaowen Wang[2]*, Cheng Jin[1]*

[1] State Key Laboratory of Mycology, Institute of Microbiology, Chinese Academy of Sciences, Beijing, 100101, China

[2] Department of Dermatology and Venerology, Peking University First Hospital; Research [3] Center for Medical Mycology, Peking University; Beijing Key Laboratory of Molecular Diagnosis on Dermatoses; National Clinical Research Center for Skin and Immune Diseases, Beijing, 100034, China

* Corresponding author's email: jinc@im.ac.cn, ouyanghm@im.ac.cn, or xiaowenpku@126.com

[#] These authors have contributed equally to this work.

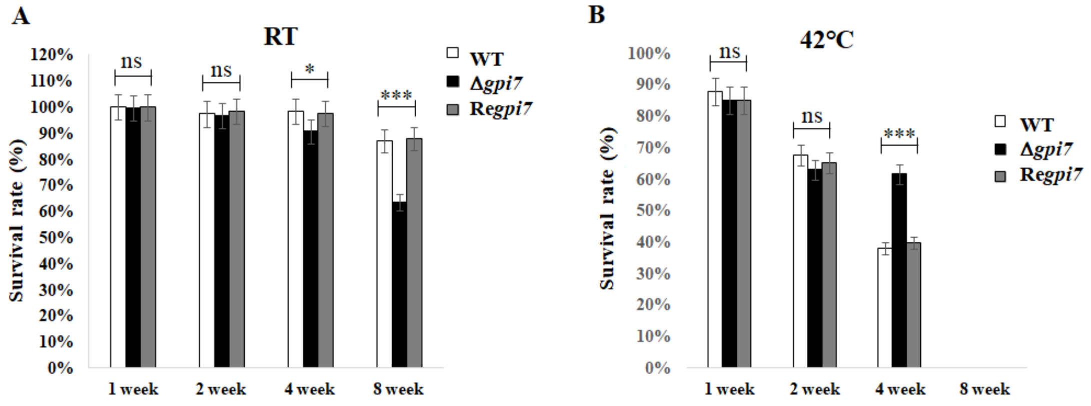

Fig.S1. Viability of the mutant conidia. 10^5 conidia were kept in distilled water for different storage time (1 week, 2 weeks, 4 weeks, and 8 weeks) at room temperature (A) or 42℃ (B). At intervals, the viable conidia were counted on CM plate. For statistical significance each experiment was performed 5 times for each strain. Mean and SD are presented. ns, not signifcant; *, $p<0.05$; ***, $p<0.001$.
